# Serum testosterone as a biomarker for second prostatic biopsy in men with negative first biopsy for prostatic cancer and PSA>4ng/mL, or with PIN biopsy result

**DOI:** 10.1590/S1677-5538.IBJU.2015.0167

**Published:** 2016

**Authors:** Alexandros Fiamegos, John Varkarakis, Michael Kontraros, Andreas Karagiannis, Michael Chrisofos, Dimitrios Barbalias, Charalampos Deliveliotis

**Affiliations:** 1 2nd Department of Urology, University of Athens, Sismanoglio General Hospital, Athens, Greece

**Keywords:** Testosterone, Prostate, Neoplasms, Biopsy

## Abstract

**Introduction:**

Data from animal, clinical and prevention studies support the role of androgens in prostate cancer growth, proliferation and progression. Results of serum based epidemiologic studies in humans, however, have been inconclusive. The present study aims to define whether serum testosterone can be used as a predictor of a positive second biopsy in males considered for re-biopsy.

**Material and Methods:**

The study included 320 men who underwent a prostatic biopsy in our department from October 2011 until June 2012. Total testosterone, free testosterone, bioavailable testosterone and prostate pathology were evaluated in all cases. Patients undergoing a second biopsy were identified and biopsy results were statistically analyzed.

**Results:**

Forty men (12.5%) were assessed with a second biopsy. The diagnosis of the second biopsy was High Grade Intraepithelial Neoplasia in 14 patients (35%) and Prostate Cancer in 12 patients (30%). The comparison of prostatic volume, total testosterone, sex hormone binding globulin, free testosterone, bioavailable testosterone and albumin showed that patients with cancer of the prostate had significantly greater levels of free testosterone (p=0.043) and bioavailable T (p=0.049).

**Conclusion:**

In our study, higher free testosterone and bioavailable testosterone levels were associated with a cancer diagnosis at re-biopsy. Our results indicate a possible role for free and bioavailable testosterone in predicting the presence of prostate cancer in patients considered for re-biopsy.

## INTRODUCTION

Prostate cancer is the second most common cancer in men worldwide (1) and the only established risk factors for prostate cancer are age, race and family history (2). Androgens seem to play a key role in the natural history of prostate cancer. In fact, androgens are required for the normal growth and development of the prostate gland and high levels of androgens have long been considered to be possible risk factors for prostate cancer (3, 4). Large doses of androgens can induce prostate cancer in rodents (5) and they also stimulate in vitro human prostate cancer cell proliferation (6). Prostate cancer presents only rarely among castrated men, while surgical or medical castration in prostate cancer patients causes tumor regression (7).

However, there are many shortcomings in using testosterone as a tool for prostate cancer diagnosis or prognosis. In fact, many observational studies failed to demonstrate any clear association between testosterone levels and risk of prostate cancer diagnosis, and the exact mechanism of action of testosterone on prostatic tissue has not been to date fully elucidated. Prostate cancer diagnosis is currently based mainly upon digital rectal examination (DRE) and/or PSA measurement leading to a transrectal prostatic ultrasound guided biopsy (TRUS-b). A problem in this regard is assessing patients after a negative biopsy, as rates of prostate cancer diagnosis fall substantially thereafter.

In the present study we aim to determine whether serum testosterone can be used as a predictor of a positive second biopsy in males considered for re-biopsy, either after a negative first biopsy, or with a high grade intra prostatic neoplasia (HGPIN) diagnosis.

## MATERIAL AND METHODS

The study included 320 consecutive men who underwent a prostatic biopsy in our department from October 2011 until June 2012. All 320 patients were informed about participating in the study and have signed a consent form with full information about the procedure, the aims of the study and the possible outcomes. The study was approved by the Medical School of University of Athens board and the ethics committee of Sismanoglion General Hospital of Athens. Decision for biopsy was based upon high serum PSA levels and/or suspicious DRE findings according to current practice as pointed out by the EAU guidelines. The serum PSA level which led a patient to a biopsy was above 4ng/mL. Only 17 out of the 320 patients were led to biopsy with clinical suspicion from the digital rectal examination and had serum PSA level lower than 4ng/mL. The biopsy was performed transrectally under ultrasound guidance using a spring-driven biopsy gun, between 07:00 and 09:00 am. Before the TRUS biopsy procedure was undertaken, a complete history was acquired, including race and ethnicity and detailed dietary habits history, as well as peripheral vein blood samples for free and total PSA (if not already performed), free testosterone (fTe), total testosterone (tTe), albumin (ALB) and sex hormone binding globulin (SHBG) levels measurement. During the TRUS biopsy, the prostatic size was measured and recorded. The biopsy was performed by one physician. The sextant cores pattern was used with 12 core biopsy. This pattern was changed based on the TRUS findings concerning the size of the prostate and possibly suspicious regions and varied from 8 cores from a small prostate to 28 cores for large prostatic glands, but most of the patients underwent a 12 cores biopsy and the number of the cores taken proved to be of no importance for the diagnosis of prostate cancer or not. Tissue samples were examined by the pathologic laboratory of our facility, by one pathologist.

The decision for a second biopsy was based on three criteria. The first one was high clinical suspicion due to digital rectal examination. The second one was repeated high PSA levels (>4ng/mL), despite a negative result from the first biopsy. The third one is HGPIN diagnosis in the initial biopsy. The second biopsy was performed within a year from the first one and the meantime between the two biopsies varied due to the different criteria for second biopsy and due to patient preferences and awareness.

Total Testosterone (tTe) and sex hormone binding globulin (SHBG) were measured using electrochemiluminescence immunoassay “ellia” for use in immunological analysis [elecsys 1010/2010 and modular analytics E170 (subunit elecsys) of Roche]. Albumin (ALB) was measured using a colorimetric assay endpoint method, and PSA with heterogeneous direct chemiluminescence (sandwich type, using two monoclonal antibodies). The free testosterone (fTe) and bioavailable testosterone (BioTe) levels were calculated implementing accepted published equations using tTe, SHBG and ALB (8, 9). We measured fTe and BioTe, as these are considered to be the actual amount of testosterone available at a cellular level (8).

### Statistical analysis

Continuous variables are presented with mean and standard deviation (SD). Quantitative variables are presented with absolute and relative frequencies. For the comparison of proportion between the two study groups chi-square and Fisher's exact tests were used. Student's t-tests were computed for the comparison of mean values. All p values reported are two-tailed. Statistical significance was set at 0.05 and analyses were conducted using SPSS statistical software (version 18.0).

## RESULTS

Sample characteristics are presented in Table-1. All the patients were Caucasians, 318 Greeks, 1 Hungarian and 1 Italian concerning the race and the ethnicity. The analysis of the detailed nutrition habits history has shown no statistical important difference between the positive and the negative for cancer group. In total, a sample of 320 patients was selected and 40 of them (12.5%) had a re-biopsy. The decision for second biopsy was based on high clinical suspicion for prostate cancer but negative first biopsy. The patients of the second biopsy were patients with high PSA levels (>4ng/mL) and negative biopsy and/or HGPIN who were informed about the options they had and agreed to undergo a second prostatic biopsy within the timeframe of our study. The same formal requirements were followed for the second biopsy. The second biopsy was performed by the same physician, was investigated by the same pathologist and the number of cores taken from the prostate were 20 for all the patients, regardless the result of the first biopsy or the number of cores taken at the first biopsy. The diagnosis of the second biopsy was HGPIN in 14 patients (35%) and CaP in 12 patients (30%). From the 14 patients with HGPIN 8 had a negative first biopsy and 6 had a HGPIN at first biopsy, while from the 12 patients with CaP, 6 had a negative first biopsy and 6 had a HGPIN result in their first biopsy. When prostatic volume (Vp), total testosterone, SHBG, free testosterone, bioavailable testosterone and albumin were compared between patients that underwent a second biopsy and those who did not, no statistically significant differences were found (Table-2). The comparison of the aforementioned indices between patients with a cancer diagnosis and those with negative or a HGPIN result in the second biopsy (Table-3) showed that patients with prostatic cancer (CaP) had significantly higher levels of free testosterone (p=0.043) and bioavailable T (p=0.049).

**Table 1:** Sample characteristics.

		Mean(SD)
**Total sample (N=320)**		
	Age	67(8.1)
	PSA ng/mL	8.3(4.3)
**Sample with 2^nd^ biopsy (N=40)**		
	Age	66.4(8.8)
	PSA ng/mL	8.0(3.7)
**2^nd^ Biopsy diagnosis, N (%)**		
	Negative	14(35.0)
	HGPIN	14(35.0)
	Prostate Cancer	12(30.0)

**SD** = Standard Deviation

**Table 2:** Comparative analysis of patients with and without rebiopsy regarding measured variables.

		2nd Biopsy		
		No	Yes	
		Number (%)	Number (%)	P
**Total Testosterone ng/dL**				
	<231 ng/dL	28(10)	1(2.5)	0.217**
	231–346 ng/dL	39(13.9)	8(20)	
	>346 ng/dL	213(76.1)	31(77.5)	
		Mean(SD)	Mean(SD)	P
Prostate Volume mL		49.7(22.5)	47.4(18.1)	0.534*
Total Testosterone ng/dL		410.9(145.2)	423.6(120.3)	0.597*
SHBG nmol/L		49.9(20.8)	50.4(18.4)	0.873*
Free Testosterone ng/dL		6.6(2.7)	6.9(3.5)	0.569*
Bioavaillable Testosterone ng/dL		161.6(65.2)	164.4(81.2)	0.807*
Albumin g/dL		4.5(0.3)	4.5(0.2)	0.640*

* Student's t-test

** Chi-square test

**SD** = Standard Deviation

**Table 3:** Comparative analysis of patients with and without CaP after rebiopsy, regarding measured variables.

		2nd Biopsy diagnosis		
		Negative/HGPIN	CaP	
		Number (%)	Number (%)	P
**Total Testosterone ng/dL**				
	<231 ng/dL	1(3.6)	0(0.0)	0.778**
	231–346 ng/dL	5(17.9)	3(25.0)	
	>346 ng/dL	22(78.6)	9(75.0)	
		Mean(SD)	Mean(SD)	P
Prostate Volume mL		45.9(16.8)	50.7(21.2)	0.451*
Total Testosterone ng/dL		409.1(100.5)	457.4(157.4)	0.250*
SHBG nmol/L		51(17.2)	49.1(21.9)	0.765*
Free Testosterone ng/dL		6.1(1.8)	8.6(5.6)	0.044*
Bioavaillable Testosterone ng/dL		148(50.2)	202.8(122.1)	0.043*
Albumin g/dL		4.5(0.2)	4.5(0.3)	0.407*

* Student's t-test

** Fisher's exact test

**SD** = Standard Deviation

## DISCUSSION

In men, testosterone is predominantly produced by Leydig cells of the testes; only a lesser amount (<10%) is produced in the adrenal glands. Testosterone plays a key role in the development of male reproductive tissues (10). It stimulates the prostate gland to grow both early in puberty, when the prostate doubles in size, and around age 25, when the gland begins to grow again. Testosterone is converted into dihydrotestosterone (DHT), which is the androgen receptor's major activator (11, 12). After DHT binds to androgen receptors, it translocates into the nucleus, where it mediates the transcriptional activation of target genes (13). Through androgen-stimulated changes in gene expression cellular growth occurs, eventually leading to benign prostatic hyperplasia in elderly men.

Great controversy has risen in the last years regarding the exact relation between prostate cancer and androgens. The first published work on this subject goes many years back in 1941, when for the first time Huggins and Hodges proved that testosterone deprivation leads to prostate cancer regression (7). Since then many studies tried to elucidate the relation between prostate cancer and testosterone. Many groups reported that the risk of prostate cancer was higher in men with lower total testosterone levels (14, 15) and bioavailable testosterone level (16). An important role seems to be played by SHBG, which in turn determines the levels of free and bioavailable testosterone. A recent study showed that there is a relation between SHBG level and prostate cancer especially in younger patients (17). Nonetheless, an article by Roddam et al. reported no association between blood levels of total testosterone and prostate cancer risk based on pooled analysis of 18 prospective studies (18). The pooled analysis included 3886 men with prostate cancer and 6438 controls. It is the largest serum based study with the most elegant and comprehensive analysis to date to test a central hypothesis in prostate cancer etiology. It is important to note, that the pooled analysis did not find a positive link between circulating levels of total testosterone and prostate cancer risk, but few of the eighteen studies included reported a substantial positive association. On the other hand, fear that androgen supplementation could cause prostate cancer arousal in males, especially after prostate biopsy findings of HGPIN, has not been proven (19).

As shown in other studies, we also found no association between serum testosterone level and prostate cancer diagnosis at biopsy (17, 20, 21). Furthermore, more recent studies continuously provide controversial results regarding serum androgens and prediction of prostate cancer. Regis et al. in their review of 124 publications came up to the conclusion that due to the heterogeneity of the studies they cannot recommend testosterone level measurement in order to predict prostate cancer and its aggressiveness (22). On the other hand, the correlation between testosterone and PSA remains (23). Additionally experimental in vitro and mice data show that testosterone might be used to help prediction of CaP in low PSA level measurements (24), as Usoro et al. showed that patients with CaP have higher estradiol levels while presenting no difference in testosterone levels (25). Focusing on this idea Black et al. suggest that the ratio estrogen/androgen may be important in the development of CaP (26).

In our study we attempt to determine, whether testosterone level is associated with a positive second prostate biopsy, both in patients that were found to harbour HGPIN at the first biopsy and those that were not. Our study cannot confirm the findings of the study by Garcia-Cruz et al., who found that patients with a positive re-biopsy after HGPIN have significantly lower free testosterone and bioavailable testosterone and higher SHBG levels than men with a negative re-biopsy (27). In fact, we determined an association between higher levels of free and bioavailable testosterone and cancer of the prostate at the second biopsy. A possible explanation of such discordance might rest on differences of cancer patient population. In our study, cancer patients at re-biopsy were found to harbour localized cancer, mostly well or moderately differentiated and only two cases of Gleason Score 7 (3+4). This was not the case in many of the studies that found an association between low testosterone (total, free or bioavailable) and prostate cancer, where a high Gleason Score was prominent (14, 20, 28–31). Low T level before a radical prostatectomy was found to carry a poor prognosis with regard to Gleason score (32). In fact, Morgentaler et al. hypothesized that prostate cancer in a low testosterone environment may dedifferentiate and give rise to undifferentiated and more aggressive tumors, and this is reflected as higher Gleason score tumors (33). His Saturation Model could explain the relationship between prostate cancer and circulating levels of testosterone (34). Additionally in order to extend this hypothesis, Pichon et al. highlight that low serum testosterone is an independent risk factor for predominant Gleason pattern 4 on prostate specimen after RP and for upgrading from low to high-grade cancer between prostate needle biopsies and RP specimen (35). With this findings seem to agree Mohit Khera et al., who claim that current evidence indicates that maximal androgen-stimulated PCa growth is achieved at relatively low serum testosterone concentrations (36). In the prostate, when all the testosterone receptors are occupied with circulating testosterone, an increase in serum testosterone level has no further physiological effect and the maximum androgen stimulation has been attained. Another possible cause for this discordance could be the fact that only patients with HGPIN were considered in the study by Garcia-Cruz, while in our study we considered patients without HGPIN as well.

There are several limitations to the present study. It is a single-centre, small series, and patient number undergoing re-biopsy was low. Moreover, we did not directly calculate the free Testosterone level. Lastly, the re-biopsy in our series occurred early, within the time span of our study (one year). The rates of cancer detection at re-biopsy rise with time, so some patients with negative biopsy might be diagnosed with cancer in the future. Larger prospective studies should be designed to elucidate the exact relation between testosterone and prostate cancer, as well as the possible use of total, free and bioavailable testosterone as a marker for prostate cancer diagnosis at the second biopsy.

## CONCLUSIONS

In our study, serum concentration of total testosterone was not associated with the risk of prostate cancer in men who underwent a second prostatic biopsy. However, higher free testosterone and bioavailable testosterone levels were associated with a cancer diagnosis at re-biopsy. Further research is required to define the complex interplay between sex steroids, genetic and lifestyle factors in prostate cancer aetiology. Our results indicate a possible role of free and bioavailable testosterone in predicting the presence of prostate cancer in patients considered for re-biopsy.
